# Chloridometh­yl(2-methyl­quinolin-8-olato-κ^2^ *N*,*O*)phenyl­tin(IV)

**DOI:** 10.1107/S1600536810027790

**Published:** 2010-07-17

**Authors:** Maryam Vafaee, Mostafa M. Amini, Seik Weng Ng

**Affiliations:** a Department of Chemistry, General Campus, Shahid Beheshti University, Tehran 1983963113, Iran; b Department of Chemistry, University of Malaya, 50603 Kuala Lumpur, Malaysia

## Abstract

The asymmetric unit of the title complex, [Sn(CH_3_)(C_6_H_5_)(C_10_H_8_NO)Cl], consists of two independent mol­ecules, both of which have the *N*,*O*-chelated Sn^IV^ atom in a *cis*-C_2_SnNOCl trigonal-bipyramidal geometry [C—Sn—C = 124.82 (8) and 137.69 (8)°]. The Cl atom of the mol­ecule with the smaller C—Sn—C angle inter­acts weakly with the Sn^IV^ atom of the mol­ecule with the wider C—Sn—C angle at an Sn⋯Cl distance of 3.595 (1) Å. Weak inter­molecular C—H⋯O and C—H⋯Cl hydrogen bonding is present in the crystal structure.

## Related literature

For a related structure, see: Vafaee *et al.* (2010 ▶).
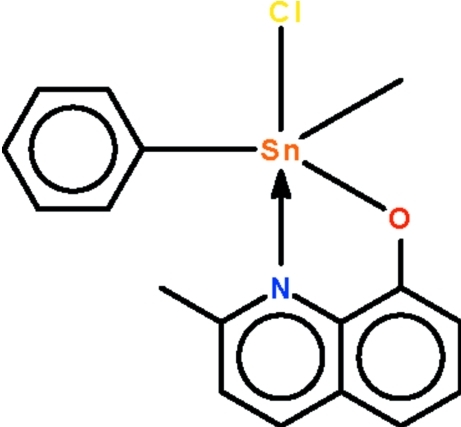


## Experimental

### 

#### Crystal data

[Sn(CH_3_)(C_6_H_5_)(C_10_H_8_NO)Cl]
*M* *_r_* = 404.45
Triclinic, 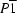

*a* = 8.9728 (4) Å
*b* = 13.1046 (6) Å
*c* = 14.0405 (6) Å
α = 106.621 (1)°
β = 92.764 (1)°
γ = 95.256 (1)°
*V* = 1570.52 (12) Å^3^
*Z* = 4
Mo *K*α radiation
μ = 1.79 mm^−1^
*T* = 100 K
0.35 × 0.35 × 0.10 mm

#### Data collection

Bruker SMART APEX diffractometer
Absorption correction: multi-scan (*SADABS*; Sheldrick, 1996 ▶) *T*_min_ = 0.572, *T*_max_ = 0.841
15157 measured reflections
7186 independent reflections
6630 reflections with *I* > 2σ(*I*)
*R*_int_ = 0.019

#### Refinement

*R*[*F* ^2^ > 2σ(*F* ^2^)] = 0.020
*wR*(*F* ^2^) = 0.057
*S* = 1.09
7186 reflections
383 parameters
H-atom parameters constrained
Δρ_max_ = 0.51 e Å^−3^
Δρ_min_ = −0.67 e Å^−3^

### 

Data collection: *APEX2* (Bruker, 2009 ▶); cell refinement: *SAINT* (Bruker, 2009 ▶); data reduction: *SAINT*; program(s) used to solve structure: *SHELXS97* (Sheldrick, 2008 ▶); program(s) used to refine structure: *SHELXL97* (Sheldrick, 2008 ▶); molecular graphics: *X-SEED* (Barbour, 2001 ▶); software used to prepare material for publication: *publCIF* (Westrip, 2010 ▶).

## Supplementary Material

Crystal structure: contains datablocks global, I. DOI: 10.1107/S1600536810027790/xu2800sup1.cif

Structure factors: contains datablocks I. DOI: 10.1107/S1600536810027790/xu2800Isup2.hkl

Additional supplementary materials:  crystallographic information; 3D view; checkCIF report

## Figures and Tables

**Table 1:** Hydrogen-bond geometry (Å, °)

*D*—H⋯*A*	*D*—H	H⋯*A*	*D*⋯*A*	*D*—H⋯*A*
C4—H4⋯Cl1^i^	0.95	2.83	3.6898 (19)	152
C22—H22⋯Cl2^i^	0.95	2.76	3.705 (2)	177
C10—H10⋯O1^ii^	0.95	2.59	3.454 (2)	152

Symmetry codes: (i) 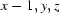; (ii) 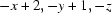.

**Fig. 1.**
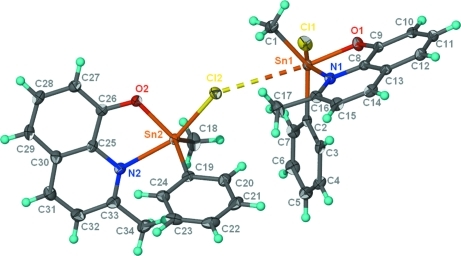
Thermal ellipsoid plot (Barbour, 2001) of the two independent molecules of SnCl(CH3)(C6H5)(C10H8NO) at the 70% probability level. Hydrogen atoms are drawn as spheres of arbitrary radius. The Cl2 atom is 3.595 (1) Å from Sn1, and it opens the C–Sn–C angle to 137.69 (8) °.

## supplementary crystallographic information

### Comment

The anion of 8-hydroxyquinoline is known to chelate to tin in organotin(IV) quinolinolates; however, for the chloroorganotin quinolinates, the chlorine atom participates in weak intermolecular bridging. Chloridomethylphenyl(quinolin-8-olato)tin exists as a dinuclear molecule owing to bridging by the anion (Vafaee *et al.*, 2010). The methyl-substituted quinolin-8-olato derivative (Scheme I) consists of two independent molecules, and both have the *N*,*O*-chelated tin(IV) atom in a *cis*-C_2_SnNOCl trigonal bipyramidal geometry. The C18–Sn2–C19 and C1–Sn1–C2 bond angles are 124.82 (8) and 137.69 (8)°, respectively. The chlorine atom (Cl2) of the molecule with the smaller C–Sn–C angle interacts weakly with the tin atom (Sn1) of the molecule with the wider C–Sn–C angle at a distance of 3.595 (1) Å (Fig. 1). Weak intermolecular C—H···O and C—H···Cl hydrogen bonding is present in the crystal structure (Table 1).

### Experimental

Methylphenyltin dichloride (0.35 g, 1 mmol) and 2-methyl-8-hydroxyquinoline (0.16 g, 1 mmol) were dissolved in methanol (10 ml) to give a faint yellow solution. The solution was set aside for the growth of crystals over a few days. Slow evaporation of methanol furnished crystals.

### Refinement

Hydrogen atoms were placed in calculated positions (C–H 0.95–0.98 Å) and included in the refinement in the riding model approximation, with *U*(H) set to 1.2–1.5*U*_eq_(C).

### Crystal data

**Table:**

[Sn(CH_3_)(C_6_H_5_)(C_10_H_8_NO)Cl]	*Z* = 4
*M**_r_* = 404.45	*F*(000) = 800
Triclinic, *P*1	*D*_x_ = 1.711 Mg m^−^^3^
Hall symbol: -P 1	Mo *K*α radiation, λ = 0.71073 Å
*a* = 8.9728 (4) Å	Cell parameters from 9971 reflections
*b* = 13.1046 (6) Å	θ = 2.6–28.3°
*c* = 14.0405 (6) Å	µ = 1.79 mm^−^^1^
α = 106.621 (1)°	*T* = 100 K
β = 92.764 (1)°	Block, yellow
γ = 95.256 (1)°	0.35 × 0.35 × 0.10 mm
*V* = 1570.52 (12) Å^3^	

### Data collection

**Table:**

Bruker SMART APEX diffractometer	7186 independent reflections
Radiation source: fine-focus sealed tube	6630 reflections with *I* > 2σ(*I*)
graphite	*R*_int_ = 0.019
ω scans	θ_max_ = 27.5°, θ_min_ = 1.5°
Absorption correction: multi-scan (*SADABS*; Sheldrick, 1996)	*h* = −11→11
*T*_min_ = 0.572, *T*_max_ = 0.841	*k* = −17→17
15157 measured reflections	*l* = −18→16

### Refinement

**Table:**

Refinement on *F*^2^	Primary atom site location: structure-invariant direct methods
Least-squares matrix: full	Secondary atom site location: difference Fourier map
*R*[*F*^2^ > 2σ(*F*^2^)] = 0.020	Hydrogen site location: inferred from neighbouring sites
*wR*(*F*^2^) = 0.057	H-atom parameters constrained
*S* = 1.09	*w* = 1/[σ^2^(*F*_o_^2^) + (0.0305*P*)^2^ + 0.4536*P*] where *P* = (*F*_o_^2^ + 2*F*_c_^2^)/3
7186 reflections	(Δ/σ)_max_ = 0.001
383 parameters	Δρ_max_ = 0.51 e Å^−^^3^
0 restraints	Δρ_min_ = −0.66 e Å^−^^3^

### Fractional atomic coordinates and isotropic or equivalent isotropic displacement parameters (Å^2^)

**Table:**

	*x*	*y*	*z*	*U*_iso_*/*U*_eq_	
Sn1	0.861340 (14)	0.615377 (9)	0.290747 (9)	0.01263 (4)	
Sn2	0.799527 (14)	0.832410 (9)	0.702160 (9)	0.01230 (4)	
Cl1	1.10698 (5)	0.72185 (4)	0.30174 (4)	0.02216 (11)	
Cl2	0.84380 (6)	0.68692 (4)	0.55666 (4)	0.01948 (10)	
O1	0.88484 (15)	0.56320 (11)	0.14165 (10)	0.0167 (3)	
O2	0.92261 (15)	0.75531 (11)	0.77964 (10)	0.0158 (3)	
N1	0.63791 (18)	0.49944 (12)	0.22228 (12)	0.0135 (3)	
N2	0.78441 (18)	0.92597 (12)	0.87127 (12)	0.0143 (3)	
C1	0.9407 (3)	0.50330 (17)	0.35798 (16)	0.0225 (4)	
H1A	0.8590	0.4744	0.3899	0.034*	
H1B	1.0232	0.5388	0.4082	0.034*	
H1C	0.9768	0.4447	0.3070	0.034*	
C2	0.7376 (2)	0.75143 (15)	0.33138 (14)	0.0136 (4)	
C3	0.5837 (2)	0.74372 (15)	0.30636 (14)	0.0154 (4)	
H3	0.5317	0.6762	0.2704	0.018*	
C4	0.5052 (2)	0.83317 (16)	0.33318 (15)	0.0186 (4)	
H4	0.4004	0.8264	0.3157	0.022*	
C5	0.5804 (2)	0.93240 (16)	0.38556 (16)	0.0200 (4)	
H5	0.5274	0.9938	0.4034	0.024*	
C6	0.7327 (2)	0.94134 (16)	0.41155 (16)	0.0205 (4)	
H6	0.7842	1.0089	0.4480	0.025*	
C7	0.8110 (2)	0.85155 (15)	0.38452 (15)	0.0181 (4)	
H7	0.9157	0.8586	0.4025	0.022*	
C8	0.6448 (2)	0.46140 (14)	0.12108 (14)	0.0128 (4)	
C9	0.7758 (2)	0.49533 (14)	0.08082 (14)	0.0131 (4)	
C10	0.7884 (2)	0.45633 (15)	−0.02044 (14)	0.0150 (4)	
H10	0.8756	0.4781	−0.0485	0.018*	
C11	0.6728 (2)	0.38445 (15)	−0.08252 (15)	0.0168 (4)	
H11	0.6850	0.3569	−0.1517	0.020*	
C12	0.5437 (2)	0.35347 (15)	−0.04558 (15)	0.0164 (4)	
H12	0.4661	0.3064	−0.0890	0.020*	
C13	0.5265 (2)	0.39227 (14)	0.05808 (14)	0.0141 (4)	
C14	0.3962 (2)	0.36966 (15)	0.10369 (15)	0.0167 (4)	
H14	0.3118	0.3265	0.0641	0.020*	
C15	0.3902 (2)	0.40939 (15)	0.20431 (15)	0.0172 (4)	
H15	0.3016	0.3944	0.2345	0.021*	
C16	0.5159 (2)	0.47280 (14)	0.26363 (14)	0.0154 (4)	
C17	0.5142 (3)	0.50856 (17)	0.37524 (15)	0.0223 (4)	
H17A	0.5913	0.5692	0.4034	0.033*	
H17B	0.5348	0.4493	0.4017	0.033*	
H17C	0.4154	0.5305	0.3934	0.033*	
C18	0.9310 (3)	0.95753 (17)	0.66836 (16)	0.0246 (5)	
H18A	0.8988	1.0265	0.7049	0.037*	
H18B	0.9184	0.9484	0.5966	0.037*	
H18C	1.0369	0.9558	0.6879	0.037*	
C19	0.5611 (2)	0.80153 (14)	0.67682 (15)	0.0143 (4)	
C20	0.5010 (2)	0.78133 (15)	0.57891 (15)	0.0184 (4)	
H20	0.5660	0.7825	0.5277	0.022*	
C21	0.3465 (2)	0.75946 (16)	0.55562 (17)	0.0225 (4)	
H21	0.3064	0.7477	0.4890	0.027*	
C22	0.2512 (2)	0.75486 (16)	0.62948 (18)	0.0237 (5)	
H22	0.1459	0.7388	0.6135	0.028*	
C23	0.3109 (2)	0.77407 (16)	0.72750 (17)	0.0225 (4)	
H23	0.2461	0.7703	0.7782	0.027*	
C24	0.4646 (2)	0.79866 (15)	0.75122 (15)	0.0172 (4)	
H24	0.5042	0.8136	0.8184	0.021*	
C25	0.8496 (2)	0.87232 (14)	0.92987 (14)	0.0135 (4)	
C26	0.9237 (2)	0.78282 (14)	0.87912 (14)	0.0135 (4)	
C27	0.9918 (2)	0.72626 (15)	0.93489 (15)	0.0165 (4)	
H27	1.0450	0.6681	0.9029	0.020*	
C28	0.9827 (2)	0.75434 (16)	1.03909 (16)	0.0191 (4)	
H28	1.0289	0.7136	1.0760	0.023*	
C29	0.9091 (2)	0.83888 (16)	1.08871 (15)	0.0195 (4)	
H29	0.9029	0.8555	1.1587	0.023*	
C30	0.8425 (2)	0.90100 (15)	1.03417 (15)	0.0169 (4)	
C31	0.7673 (3)	0.99163 (17)	1.07720 (16)	0.0224 (4)	
H31	0.7585	1.0144	1.1471	0.027*	
C32	0.7074 (3)	1.04612 (16)	1.01742 (16)	0.0238 (5)	
H32	0.6591	1.1079	1.0465	0.029*	
C33	0.7165 (2)	1.01183 (15)	0.91330 (16)	0.0183 (4)	
C34	0.6501 (3)	1.07147 (16)	0.84782 (16)	0.0236 (5)	
H34A	0.6122	1.0208	0.7834	0.035*	
H34B	0.7274	1.1243	0.8374	0.035*	
H34C	0.5673	1.1083	0.8799	0.035*	

### Atomic displacement parameters (Å^2^)

**Table:**

	*U*^11^	*U*^22^	*U*^33^	*U*^12^	*U*^13^	*U*^23^
Sn1	0.01233 (7)	0.01319 (7)	0.01122 (7)	0.00009 (5)	0.00001 (5)	0.00230 (5)
Sn2	0.01178 (7)	0.01214 (7)	0.01252 (7)	−0.00020 (5)	−0.00045 (5)	0.00358 (5)
Cl1	0.0134 (2)	0.0269 (2)	0.0216 (2)	−0.00386 (18)	0.00076 (19)	0.0016 (2)
Cl2	0.0179 (2)	0.0228 (2)	0.0148 (2)	0.00503 (18)	−0.00028 (18)	0.00032 (18)
O1	0.0135 (7)	0.0210 (7)	0.0127 (7)	−0.0030 (5)	−0.0001 (5)	0.0020 (5)
O2	0.0170 (7)	0.0169 (6)	0.0134 (7)	0.0048 (5)	0.0000 (5)	0.0039 (5)
N1	0.0143 (8)	0.0120 (7)	0.0134 (8)	−0.0001 (6)	−0.0004 (6)	0.0029 (6)
N2	0.0139 (8)	0.0129 (7)	0.0150 (8)	−0.0003 (6)	−0.0014 (6)	0.0033 (6)
C1	0.0281 (12)	0.0199 (10)	0.0209 (10)	0.0087 (8)	0.0003 (9)	0.0066 (8)
C2	0.0159 (9)	0.0151 (8)	0.0106 (8)	0.0011 (7)	0.0016 (7)	0.0053 (7)
C3	0.0177 (10)	0.0154 (9)	0.0129 (9)	−0.0011 (7)	−0.0019 (7)	0.0053 (7)
C4	0.0166 (10)	0.0205 (9)	0.0211 (10)	0.0027 (8)	−0.0005 (8)	0.0099 (8)
C5	0.0250 (11)	0.0164 (9)	0.0210 (10)	0.0049 (8)	0.0034 (8)	0.0080 (8)
C6	0.0232 (11)	0.0142 (9)	0.0219 (10)	−0.0026 (8)	0.0009 (8)	0.0031 (8)
C7	0.0170 (10)	0.0168 (9)	0.0187 (10)	−0.0025 (7)	0.0010 (8)	0.0038 (8)
C8	0.0138 (9)	0.0107 (8)	0.0139 (9)	0.0029 (7)	−0.0002 (7)	0.0031 (7)
C9	0.0140 (9)	0.0127 (8)	0.0120 (9)	0.0022 (7)	−0.0007 (7)	0.0028 (7)
C10	0.0125 (9)	0.0189 (9)	0.0146 (9)	0.0037 (7)	0.0017 (7)	0.0057 (7)
C11	0.0205 (10)	0.0160 (9)	0.0125 (9)	0.0053 (7)	−0.0008 (7)	0.0013 (7)
C12	0.0175 (10)	0.0134 (8)	0.0160 (9)	0.0006 (7)	−0.0038 (8)	0.0018 (7)
C13	0.0151 (9)	0.0105 (8)	0.0161 (9)	0.0026 (7)	−0.0008 (7)	0.0030 (7)
C14	0.0149 (9)	0.0126 (8)	0.0215 (10)	−0.0025 (7)	−0.0040 (8)	0.0055 (7)
C15	0.0164 (10)	0.0155 (9)	0.0210 (10)	−0.0015 (7)	0.0025 (8)	0.0086 (8)
C16	0.0177 (10)	0.0117 (8)	0.0166 (9)	0.0005 (7)	0.0031 (8)	0.0041 (7)
C17	0.0251 (11)	0.0218 (10)	0.0180 (10)	−0.0035 (8)	0.0054 (8)	0.0040 (8)
C18	0.0296 (12)	0.0213 (10)	0.0218 (11)	−0.0083 (9)	0.0000 (9)	0.0084 (8)
C19	0.0130 (9)	0.0103 (8)	0.0183 (9)	0.0005 (7)	−0.0015 (7)	0.0026 (7)
C20	0.0198 (10)	0.0158 (9)	0.0195 (10)	0.0021 (7)	−0.0016 (8)	0.0055 (8)
C21	0.0195 (11)	0.0199 (10)	0.0255 (11)	0.0028 (8)	−0.0081 (9)	0.0041 (8)
C22	0.0123 (10)	0.0190 (9)	0.0360 (12)	0.0021 (8)	−0.0020 (9)	0.0023 (9)
C23	0.0174 (10)	0.0168 (9)	0.0314 (12)	0.0027 (8)	0.0072 (9)	0.0029 (8)
C24	0.0179 (10)	0.0138 (8)	0.0186 (10)	0.0022 (7)	0.0014 (8)	0.0023 (7)
C25	0.0130 (9)	0.0119 (8)	0.0145 (9)	−0.0027 (7)	−0.0030 (7)	0.0041 (7)
C26	0.0100 (9)	0.0134 (8)	0.0166 (9)	−0.0023 (7)	−0.0001 (7)	0.0050 (7)
C27	0.0154 (10)	0.0158 (9)	0.0189 (10)	0.0013 (7)	0.0004 (8)	0.0063 (7)
C28	0.0192 (10)	0.0190 (9)	0.0203 (10)	−0.0002 (8)	−0.0037 (8)	0.0094 (8)
C29	0.0237 (11)	0.0209 (9)	0.0127 (9)	−0.0016 (8)	−0.0025 (8)	0.0046 (7)
C30	0.0187 (10)	0.0151 (9)	0.0148 (9)	−0.0010 (7)	−0.0014 (8)	0.0021 (7)
C31	0.0275 (12)	0.0201 (10)	0.0157 (10)	0.0037 (8)	−0.0014 (8)	−0.0009 (8)
C32	0.0280 (12)	0.0176 (9)	0.0212 (11)	0.0072 (8)	−0.0013 (9)	−0.0024 (8)
C33	0.0180 (10)	0.0122 (8)	0.0224 (10)	0.0014 (7)	−0.0038 (8)	0.0026 (7)
C34	0.0309 (12)	0.0163 (9)	0.0235 (11)	0.0086 (8)	−0.0032 (9)	0.0048 (8)

### Geometric parameters (Å, °)

**Table:**

Sn1—C1	2.116 (2)	C14—C15	1.364 (3)
Sn1—C2	2.140 (2)	C14—H14	0.9500
Sn1—N1	2.382 (2)	C15—C16	1.414 (3)
Sn1—O1	2.036 (1)	C15—H15	0.9500
Sn1—Cl1	2.4717 (5)	C16—C17	1.503 (3)
Sn2—C18	2.110 (2)	C17—H17A	0.9800
Sn2—C19	2.134 (2)	C17—H17B	0.9800
Sn2—N2	2.357 (2)	C17—H17C	0.9800
Sn2—O2	2.035 (1)	C18—H18A	0.9800
Sn2—Cl2	2.4403 (5)	C18—H18B	0.9800
O1—C9	1.343 (2)	C18—H18C	0.9800
O2—C26	1.338 (2)	C19—C20	1.394 (3)
N1—C16	1.328 (3)	C19—C24	1.395 (3)
N1—C8	1.371 (2)	C20—C21	1.393 (3)
N2—C33	1.329 (2)	C20—H20	0.9500
N2—C25	1.369 (2)	C21—C22	1.387 (3)
C1—H1A	0.9800	C21—H21	0.9500
C1—H1B	0.9800	C22—C23	1.397 (3)
C1—H1C	0.9800	C22—H22	0.9500
C2—C7	1.395 (3)	C23—C24	1.389 (3)
C2—C3	1.395 (3)	C23—H23	0.9500
C3—C4	1.392 (3)	C24—H24	0.9500
C3—H3	0.9500	C25—C30	1.410 (3)
C4—C5	1.390 (3)	C25—C26	1.427 (3)
C4—H4	0.9500	C26—C27	1.380 (3)
C5—C6	1.383 (3)	C27—C28	1.411 (3)
C5—H5	0.9500	C27—H27	0.9500
C6—C7	1.394 (3)	C28—C29	1.374 (3)
C6—H6	0.9500	C28—H28	0.9500
C7—H7	0.9500	C29—C30	1.417 (3)
C8—C9	1.419 (3)	C29—H29	0.9500
C8—C13	1.416 (3)	C30—C31	1.416 (3)
C9—C10	1.380 (3)	C31—C32	1.368 (3)
C10—C11	1.409 (3)	C31—H31	0.9500
C10—H10	0.9500	C32—C33	1.410 (3)
C11—C12	1.367 (3)	C32—H32	0.9500
C11—H11	0.9500	C33—C34	1.501 (3)
C12—C13	1.419 (3)	C34—H34A	0.9800
C12—H12	0.9500	C34—H34B	0.9800
C13—C14	1.409 (3)	C34—H34C	0.9800
			
O1—Sn1—C1	108.71 (7)	C15—C14—H14	119.8
O1—Sn1—C2	112.83 (6)	C13—C14—H14	119.8
C1—Sn1—C2	137.69 (8)	C14—C15—C16	120.02 (18)
O1—Sn1—N1	75.16 (5)	C14—C15—H15	120.0
C1—Sn1—N1	91.64 (7)	C16—C15—H15	120.0
C2—Sn1—N1	91.31 (6)	N1—C16—C15	120.90 (18)
O1—Sn1—Cl1	85.61 (4)	N1—C16—C17	119.13 (18)
C1—Sn1—Cl1	95.99 (7)	C15—C16—C17	119.96 (18)
C2—Sn1—Cl1	94.87 (5)	C16—C17—H17A	109.5
N1—Sn1—Cl1	160.71 (4)	C16—C17—H17B	109.5
O2—Sn2—C18	112.03 (8)	H17A—C17—H17B	109.5
O2—Sn2—C19	122.67 (7)	C16—C17—H17C	109.5
C18—Sn2—C19	124.82 (8)	H17A—C17—H17C	109.5
O2—Sn2—N2	74.98 (5)	H17B—C17—H17C	109.5
C18—Sn2—N2	95.47 (7)	Sn2—C18—H18A	109.5
C19—Sn2—N2	91.97 (7)	Sn2—C18—H18B	109.5
O2—Sn2—Cl2	84.65 (4)	H18A—C18—H18B	109.5
C18—Sn2—Cl2	98.01 (6)	Sn2—C18—H18C	109.5
C19—Sn2—Cl2	93.80 (5)	H18A—C18—H18C	109.5
N2—Sn2—Cl2	158.67 (4)	H18B—C18—H18C	109.5
C9—O1—Sn1	119.74 (12)	C20—C19—C24	119.20 (18)
C26—O2—Sn2	119.64 (11)	C20—C19—Sn2	116.76 (15)
C16—N1—C8	119.70 (17)	C24—C19—Sn2	124.03 (14)
C16—N1—Sn1	131.88 (13)	C21—C20—C19	120.5 (2)
C8—N1—Sn1	108.34 (12)	C21—C20—H20	119.7
C33—N2—C25	119.66 (17)	C19—C20—H20	119.7
C33—N2—Sn2	130.84 (14)	C22—C21—C20	120.1 (2)
C25—N2—Sn2	109.28 (12)	C22—C21—H21	119.9
Sn1—C1—H1A	109.5	C20—C21—H21	119.9
Sn1—C1—H1B	109.5	C21—C22—C23	119.53 (19)
H1A—C1—H1B	109.5	C21—C22—H22	120.2
Sn1—C1—H1C	109.5	C23—C22—H22	120.2
H1A—C1—H1C	109.5	C24—C23—C22	120.4 (2)
H1B—C1—H1C	109.5	C24—C23—H23	119.8
C7—C2—C3	118.14 (17)	C22—C23—H23	119.8
C7—C2—Sn1	119.86 (14)	C23—C24—C19	120.24 (19)
C3—C2—Sn1	122.00 (13)	C23—C24—H24	119.9
C4—C3—C2	121.18 (18)	C19—C24—H24	119.9
C4—C3—H3	119.4	N2—C25—C30	122.83 (17)
C2—C3—H3	119.4	N2—C25—C26	116.11 (17)
C5—C4—C3	119.90 (19)	C30—C25—C26	121.05 (17)
C5—C4—H4	120.1	O2—C26—C27	122.30 (17)
C3—C4—H4	120.1	O2—C26—C25	119.32 (17)
C6—C5—C4	119.67 (19)	C27—C26—C25	118.38 (17)
C6—C5—H5	120.2	C26—C27—C28	120.38 (18)
C4—C5—H5	120.2	C26—C27—H27	119.8
C5—C6—C7	120.25 (19)	C28—C27—H27	119.8
C5—C6—H6	119.9	C29—C28—C27	121.87 (18)
C7—C6—H6	119.9	C29—C28—H28	119.1
C2—C7—C6	120.86 (19)	C27—C28—H28	119.1
C2—C7—H7	119.6	C28—C29—C30	119.20 (18)
C6—C7—H7	119.6	C28—C29—H29	120.4
N1—C8—C9	117.15 (17)	C30—C29—H29	120.4
N1—C8—C13	122.24 (17)	C25—C30—C29	119.07 (18)
C9—C8—C13	120.61 (17)	C25—C30—C31	116.50 (18)
O1—C9—C10	121.69 (17)	C29—C30—C31	124.43 (19)
O1—C9—C8	119.60 (17)	C32—C31—C30	119.56 (19)
C10—C9—C8	118.71 (17)	C32—C31—H31	120.2
C9—C10—C11	120.56 (18)	C30—C31—H31	120.2
C9—C10—H10	119.7	C31—C32—C33	120.92 (19)
C11—C10—H10	119.7	C31—C32—H32	119.5
C12—C11—C10	121.57 (18)	C33—C32—H32	119.5
C12—C11—H11	119.2	N2—C33—C32	120.46 (19)
C10—C11—H11	119.2	N2—C33—C34	118.72 (18)
C11—C12—C13	119.45 (18)	C32—C33—C34	120.82 (18)
C11—C12—H12	120.3	C33—C34—H34A	109.5
C13—C12—H12	120.3	C33—C34—H34B	109.5
C14—C13—C8	116.55 (18)	H34A—C34—H34B	109.5
C14—C13—C12	124.39 (18)	C33—C34—H34C	109.5
C8—C13—C12	119.03 (18)	H34A—C34—H34C	109.5
C15—C14—C13	120.41 (18)	H34B—C34—H34C	109.5
			
C1—Sn1—O1—C9	86.63 (15)	C9—C8—C13—C12	2.8 (3)
C2—Sn1—O1—C9	−85.08 (14)	C11—C12—C13—C14	177.05 (18)
N1—Sn1—O1—C9	−0.01 (13)	C11—C12—C13—C8	−0.7 (3)
Cl1—Sn1—O1—C9	−178.52 (13)	C8—C13—C14—C15	−3.0 (3)
C18—Sn2—O2—C26	96.62 (15)	C12—C13—C14—C15	179.17 (18)
C19—Sn2—O2—C26	−75.80 (15)	C13—C14—C15—C16	−0.6 (3)
N2—Sn2—O2—C26	6.74 (13)	C8—N1—C16—C15	−2.7 (3)
Cl2—Sn2—O2—C26	−166.89 (13)	Sn1—N1—C16—C15	173.62 (13)
O1—Sn1—N1—C16	−177.13 (18)	C8—N1—C16—C17	175.68 (17)
C1—Sn1—N1—C16	73.95 (18)	Sn1—N1—C16—C17	−8.0 (3)
C2—Sn1—N1—C16	−63.84 (17)	C14—C15—C16—N1	3.7 (3)
Cl1—Sn1—N1—C16	−172.62 (12)	C14—C15—C16—C17	−174.72 (18)
O1—Sn1—N1—C8	−0.49 (11)	O2—Sn2—C19—C20	−129.96 (13)
C1—Sn1—N1—C8	−109.41 (13)	C18—Sn2—C19—C20	58.60 (17)
C2—Sn1—N1—C8	112.81 (12)	N2—Sn2—C19—C20	156.66 (14)
Cl1—Sn1—N1—C8	4.0 (2)	Cl2—Sn2—C19—C20	−43.89 (14)
O2—Sn2—N2—C33	178.56 (19)	O2—Sn2—C19—C24	48.84 (17)
C18—Sn2—N2—C33	67.19 (19)	C18—Sn2—C19—C24	−122.60 (16)
C19—Sn2—N2—C33	−58.07 (18)	N2—Sn2—C19—C24	−24.54 (16)
Cl2—Sn2—N2—C33	−163.77 (13)	Cl2—Sn2—C19—C24	134.91 (15)
O2—Sn2—N2—C25	−6.99 (12)	C24—C19—C20—C21	0.6 (3)
C18—Sn2—N2—C25	−118.37 (14)	Sn2—C19—C20—C21	179.43 (14)
C19—Sn2—N2—C25	116.37 (13)	C19—C20—C21—C22	−1.7 (3)
Cl2—Sn2—N2—C25	10.7 (2)	C20—C21—C22—C23	1.1 (3)
O1—Sn1—C2—C7	−109.70 (15)	C21—C22—C23—C24	0.6 (3)
C1—Sn1—C2—C7	82.00 (19)	C22—C23—C24—C19	−1.7 (3)
N1—Sn1—C2—C7	175.87 (15)	C20—C19—C24—C23	1.1 (3)
Cl1—Sn1—C2—C7	−22.43 (15)	Sn2—C19—C24—C23	−177.64 (14)
O1—Sn1—C2—C3	70.31 (16)	C33—N2—C25—C30	3.0 (3)
C1—Sn1—C2—C3	−97.99 (18)	Sn2—N2—C25—C30	−172.16 (15)
N1—Sn1—C2—C3	−4.12 (16)	C33—N2—C25—C26	−178.34 (17)
Cl1—Sn1—C2—C3	157.58 (15)	Sn2—N2—C25—C26	6.5 (2)
C7—C2—C3—C4	0.2 (3)	Sn2—O2—C26—C27	173.01 (14)
Sn1—C2—C3—C4	−179.81 (15)	Sn2—O2—C26—C25	−5.7 (2)
C2—C3—C4—C5	0.2 (3)	N2—C25—C26—O2	−1.5 (3)
C3—C4—C5—C6	−0.6 (3)	C30—C25—C26—O2	177.18 (17)
C4—C5—C6—C7	0.6 (3)	N2—C25—C26—C27	179.74 (17)
C3—C2—C7—C6	−0.2 (3)	C30—C25—C26—C27	−1.6 (3)
Sn1—C2—C7—C6	179.83 (15)	O2—C26—C27—C28	−176.31 (18)
C5—C6—C7—C2	−0.2 (3)	C25—C26—C27—C28	2.4 (3)
C16—N1—C8—C9	178.02 (17)	C26—C27—C28—C29	−1.1 (3)
Sn1—N1—C8—C9	0.90 (19)	C27—C28—C29—C30	−1.1 (3)
C16—N1—C8—C13	−1.2 (3)	N2—C25—C30—C29	178.00 (18)
Sn1—N1—C8—C13	−178.32 (14)	C26—C25—C30—C29	−0.6 (3)
Sn1—O1—C9—C10	−178.73 (13)	N2—C25—C30—C31	−1.9 (3)
Sn1—O1—C9—C8	0.5 (2)	C26—C25—C30—C31	179.53 (18)
N1—C8—C9—O1	−1.0 (3)	C28—C29—C30—C25	1.9 (3)
C13—C8—C9—O1	178.21 (16)	C28—C29—C30—C31	−178.2 (2)
N1—C8—C9—C10	178.27 (16)	C25—C30—C31—C32	−0.4 (3)
C13—C8—C9—C10	−2.5 (3)	C29—C30—C31—C32	179.8 (2)
O1—C9—C10—C11	179.46 (17)	C30—C31—C32—C33	1.5 (3)
C8—C9—C10—C11	0.2 (3)	C25—N2—C33—C32	−1.8 (3)
C9—C10—C11—C12	1.9 (3)	Sn2—N2—C33—C32	172.16 (15)
C10—C11—C12—C13	−1.6 (3)	C25—N2—C33—C34	178.06 (18)
N1—C8—C13—C14	4.0 (3)	Sn2—N2—C33—C34	−8.0 (3)
C9—C8—C13—C14	−175.16 (16)	C31—C32—C33—N2	−0.4 (3)
N1—C8—C13—C12	−178.03 (16)	C31—C32—C33—C34	179.7 (2)

### Hydrogen-bond geometry (Å, °)

**Table:**

*D*—H···*A*	*D*—H	H···*A*	*D*···*A*	*D*—H···*A*
C4—H4···Cl1^i^	0.95	2.83	3.6898 (19)	152
C22—H22···Cl2^i^	0.95	2.76	3.705 (2)	177
C10—H10···O1^ii^	0.95	2.59	3.454 (2)	152

Symmetry codes: (i) *x*−1, *y*, *z*; (ii) −*x*+2, −*y*+1, −*z*.
